# Molecular Dynamics Simulations of the Interactions between a Hydrolyzed Polyacrylamide with the Face and Edge Surfaces of Molybdenite

**DOI:** 10.3390/polym14173680

**Published:** 2022-09-05

**Authors:** Luver Echeverry-Vargas, Darwin Estrada, Leopoldo Gutierrez

**Affiliations:** 1 Department of Metallurgical Engineering, Universidad de Concepción, Concepción 4070371, Chile; 2 Water Research Center for Agriculture and Mining (CRHIAM), Universidad de Concepción, Concepción 4070411, Chile

**Keywords:** molybdenite, polyacrylamides, flotation

## Abstract

Process water used in mineral processing operations corresponds to water recovered from the thickeners and tailings dams, containing residual reagents such as hydrolyzed polyacrylamides (HPAMs). These polymers depress the flotation of different minerals, and their effect on molybdenite has been experimentally demonstrated. The objective of this work was to study the interactions between a segment of a HPAM with the face and edge of molybdenite. The sigma profile, the radial distribution functions of the HPAM, and the orientation and atomic density profiles of water molecules on the face and edge surfaces of molybdenite were calculated. The results obtained from molecular dynamics simulations showed that the interactions between the HPAM and molybdenite are mainly explained by the interactions of the amide group with the faces and edges of the mineral. Molecular dynamics simulations also showed that the HPAM molecule rearranges in such a way that the amide group moves towards the molybdenite face or edge, and the carboxylate group moves away from the mineral surface. The results obtained in the simulations showed that the interactions of the HPAM with the molybdenite edge are slightly stronger than the interaction of this molecule with the mineral face. Simulations demonstrated that the presence of the sodium and hydroxide ions reduces the concentration of HPAM around the face and edge surfaces, which is expected to affect HPAM adsorption on molybdenite. The conclusions obtained through molecular dynamics simulations are in line with the results obtained in previous studies carried out at a macroscopic scale, which reported that HPAMs adsorb onto molybdenite particles and reduce their hydrophobicity.

## 1. Introduction

Water availability in mineral processing plants has drastically decreased in arid mining areas [1,2], which makes it necessary to reuse the process water recovered from the thickeners and tailings dams. As a result, high concentrations of residual reagents dissolved in the process water are recirculated to grinding and flotation. It is well known that the efficiency of the froth flotation process depends on the physicochemical characteristics of the process water, as was previously demonstrated in different studies related to flotation in high salinity aqueous media [3,4].

Molybdenum is a strategic metal which is found together with copper sulfides in the porphyry copper deposits, and molybdenite (MoS_2_) is the main molybdenum-containing mineral [5,6,7,8,9,10]. Molybdenite particles, when dispersed in flotation slurries, occur as anisotropic entities with non-polar hydrophobic surfaces, referred to as faces, and polar hydrophilic surfaces known as edges [11]. The surface properties of molybdenite come from its crystallochemical structure made up of layers of S–Mo–S, with the S and Mo atoms connected by covalent bonds, and the S–Mo–S layers bonded by weak van der Walls forces [11]. The faces and edges are negatively charged, with the latter being more chemically active due to the interactions of molybdenum with water, forming the HMoO_4_^−^ and MoO_4_^2−^ species. The faces are less active than the edges, but defects in the form of nano-edges create hydroxylated metallic sites for the adsorption of all types of molecules [12]. Similar to other naturally hydrophobic materials [13], molybdenite flotation is strongly affected by the presence of polymers in solution [7]. Polyacrylamide (PAMs) polymers are used as flocculants in copper processing plants to improve the settling rates of tailings in the thickening stages; consequently, residual PAMs dissolved in the process water are recycled to flotation, affecting the efficiency of the process. Previous studies showed that PAMs depress the flotation of different minerals [6,14,15], and their effect on molybdenite was reported [7].

As was previously mentioned, different polymers of high molecular weight based on PAMs are effectively used as flocculants in the copper processing plants [7]. The main functional group of PAMs is the amide group (R–CHCONH_2_), and for industrial applications they are modified to produce hydrolyzed PAMs (HPAMs), which contain a series of acrylic units along the main chain such as carboxylic (R–COOH^−^), sulfonic (R–SOO^−^) and amine (R–NHH^+^) groups, which render PAMs weakly to moderately anionic or cationic [7]. These modifications are responsible for the stretching phenomenon of the PAM molecules, which induces the bridging action on the mineral particles. The number of hydrolyzed units of the HPAMs establishes the degree of anionicity (DA) of the polymer, which can be calculated by dividing the moles of the acrylates ions *m* by the total moles of the acrylate ions *m* plus the moles of the acrylamide units *n*. The presence of mechanically degraded HPAMs by the action of the pumps used to recirculate process water to flotation can also affect the efficiency of molybdenite flotation [6,7]. Therefore, the interaction of the HPAMs with molybdenite particles in the flotation process is currently of interest.

The literature related to the interactions between HPAMs and molybdenite is restricted to studies in which the topic is assessed from an experimental perspective. However, to better understand the mechanisms that explain the interactions between HPAMs molecules and molybdenite, it is necessary to work in modeling these interactions at a molecular level. Regarding this topic, Jin et al. (2014) [16] used a molecular dynamics simulation to study the interactions between water molecules and the face and edge surfaces of molybdenite. Zhang et al. [17] studied the parameters for the interactions between the molybdenite faces and water using different water models. Other researchers also studied the molecular conformation of HPAMs under aqueous media of different compositions, which is expected to affect their interactions with molybdenite [9,18]. Despite all these advances, it is still necessary to study the interactions between HPAMs and molybdenite at a molecular scale.

The objective of this work was to study the interactions between a segment of a HPAM with the face and edge of molybdenite in a 0.01 M NaCl aqueous solution. The sigma profile, the radial distribution functions of the HPAM, and the orientation and atomic density profiles of water molecules on the face and edge surfaces of molybdenite were calculated.

## 2. Methodology

### 2.1. COSMO-RS Calculation

The conductor-like screening model for realistic solvents (COMSO-RS) was used to calculate the thermodynamic properties of the studied HPAM. Quantum-based equilibrium thermodynamic methods can be used to describe the molecular behavior of polymers in aqueous solutions [19], which have been shown to produce good qualitative and quantitative predictions [20,21]. The central idea of COSMO-RS is to create a virtual conductor that introduces molecules into an aqueous medium using continuous solvation models to determine its charge distribution [19]. Using the charge distribution in the form of sigma profiles which describe molecules polarity, the COSMO-RS qualitatively identified the regions in which hydrogen bonds take place. In sigma profile graphs, each peak corresponds to the charge density of an atom, constituting the molecule. When a constituent atom presents a positive partial charge, it is screened as a negative charge density, and vice versa [19]. To obtain the sigma profiles, the geometry of the HPAM section was created and optimized using the density functional theory electronic structure program DMol3 [22,23], with graphic visualization generated through Materials Studio 2017 (Accelrys Software Inc., San Diego, CA, USA). Once the flocculant section was optimized, the Becke–Perdew [24] version of the functional Volsko–Wilk–Nusair [25] was used to obtain a better characterization of the charge distribution [26]. Once the data were calculated, the sigma profiles of the flocculant sections were generated.

### 2.2. Molecular Dynamics Simulation

The interactions between two HPAM molecules in a 0.01 M NaCl solution and the face and edge surfaces of molybdenite were studied using molecular dynamics simulations with the package simulation system LAMMPS (Large-scale Atomic/Molecular Massively Parallel Simulator) [27]. Simulations were carried out at different concentrations of NaOH, and at 298 K. The visualization system used was the VMD 1.9.3 [28], and the temperature and pressure were kept constant using a Nose–Hoover thermostat and barostat [29].

The HPAM solution models were created using the Materials studio 2017 package (Accelrys Software Inc., San Diego, CA, USA). Then, 8% of the amide monomer of a PAM section was randomly replaced by a sodium carboxylate monomer to convert the PAM chain in HPAM (DA = 8%). In this work, HPAM molecules were modeled with the OPLS-AA force field (all-atom optimized potentials for liquid simulations) [30], and the SPC/E water model was used to describe the behavior of water [31]. The Lennard–Jones parameters 12–6 of Li and Mertz [32] were used for all ions.

The force field parameters of the atoms that are part of the molybdenite crystal were taken from the universal force field (UFF) [33]. The partial atomic charges of the molybdenum (Mo) and sulfur (S) atoms corresponded to the Mulliken charges that were determined from DFT calculations of a well-defined unit cell of molybdenite using the Perdew–Wang-91 function [34], and the generalized gradient approximation [35]. The force field parameters for molybdenite are listed in Table 1. The crystal structure of molybdenite used in this study was taken from the Crystallography Open Database (COD) [36]. The lattice parameters of molybdenite were all from X-ray diffraction (XRD) measurements of natural crystals. To study the interactions of the HPAM section with the face (001) and edge (121) of molybdenite, a S–Mo–S layer with basal dimensions L_x_ × L_y_ of 7.7 × 7.75 nm^2^ and a thickness of 1.54 nm was considered. Three layers were used in the simulations to minimize the interactions of the bottom layers with the water molecules absorbed on the top layer [17].

**Figure 1:**
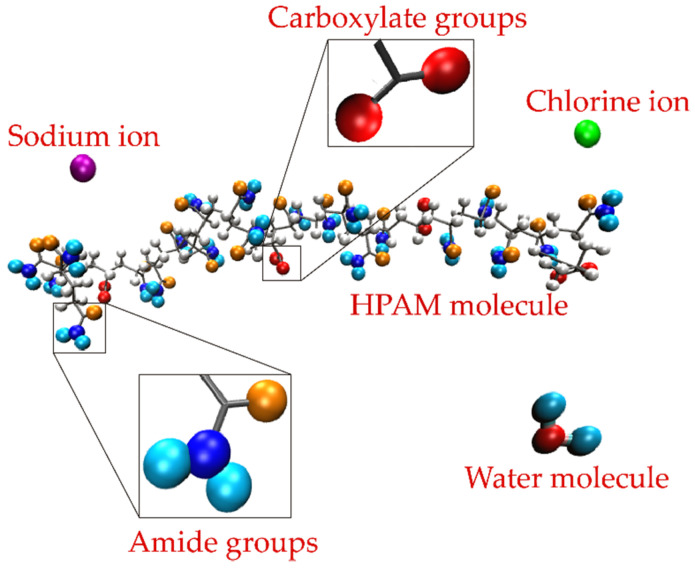
HPAM molecule indicating the areas where the carboxylate and amide groups are inserted. The water molecules and the sodium and chlorine ions are also described.

The cross section of the simulation boxes corresponded to the L_x_ × L_y_ dimensions of the molybdenite substrate with a height of 7.65 nm measured from the mineral surface. All the molybdenite atoms were constrained (frozen) in the simulations. Periodic boundary conditions were applied in the three directions of the simulation box. The HPAM molecules were embedded in the simulation box close to the molybdenite surface, then the ions were distributed at random positions maintaining a separation distance of 0.3 nm between all the atoms. Finally, 12,000 water molecules were added to the system. Figure 1 shows the simulated HPAM molecule in which the molecules of the carboxylate and amide groups, water, and sodium and chlorine ions are distinguished.

**Figure 2:**
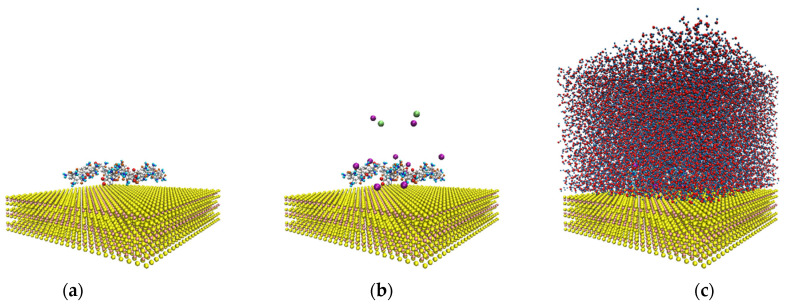
Molecular dynamics simulation setup. (**a**) Molybdenite face with two hydrolyzed polyacrylamide (HPAM) molecules; (**b**) molybdenite face with two HPAM molecules and sodium and chlorine ions; (**c**) molybdenite face with two HPAM molecules, sodium and chlorine ions, and water molecules.

Figure 2 shows the configuration of the system to study the interactions of the HPAM with the molybdenite face. Initially, the system was simulated in a NVT configuration (a constant number of particles N, volume V, and temperature T) for 100 ps at 298 K; then, the production step was carried out in a simulation NVE configuration (a constant number of particles N, volume V, and energy E) for 10 ns at 298 K, and the data were collected every 20 ps.

## 3. Results and Discussion

### 3.1. Analysis of Sigma Profile

In the COSMO-RS, sigma profiles provide essential information about an ionic compound’s tendency to make and accept hydrogen bonds [37]. In this work, the cutting region considered for hydrogen bond accepting is σ_HB < −0.0084 e/Å^2^, and for hydrogen bond donning is σ_HB > +0.0084 e/Å^2^. The sigma profiles of the molecules found within this region are non-polar in nature [37,38,39].

**Figure 3:**
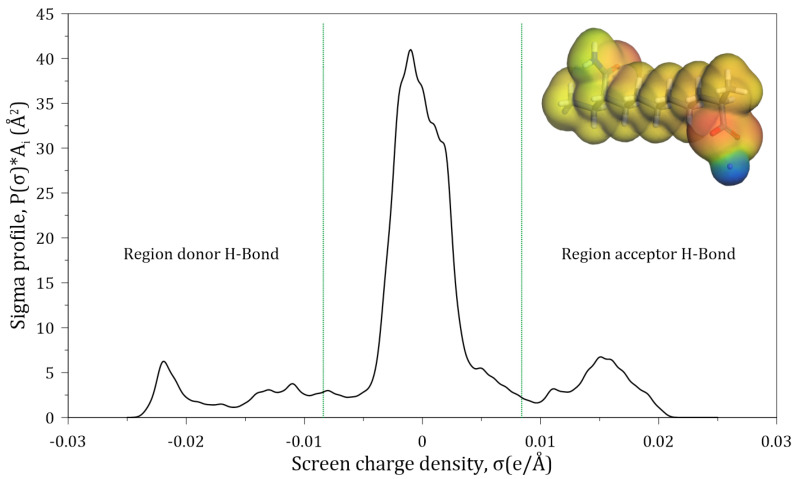
Sigma profile of a HPAM segment surface.

Figure 3 shows the sigma profile of the studied HPAM. The red color on the molecule surface corresponds to the positive surface charge which arises from the negative partial charges of the atoms within the molecule; the blue color corresponds to the negative surface charge resulting from the positive partial charge within the region; and the green and yellow colors correspond to regions of neutral charge. Figure 3 shows that a section of the HPAM is non-polar in nature and another significant part is found in the regions that are acceptors and donors of hydrogen bonds. The section of the molecule that accepts hydrogen bonds corresponds to the area in which the oxygen atoms belonging to the carboxylate and amide groups of HPAM are located; on the other hand, the hydrogen bond donor section is mainly explained by the presence of sodium ions, and to a lesser extent by the hydrogens of the amide group; the non-polar section of the HPAM corresponds to the hydrogens of the carbon chain.

### 3.2. Interactions between HPAM and Aqueous Medium

**Figure 4:**
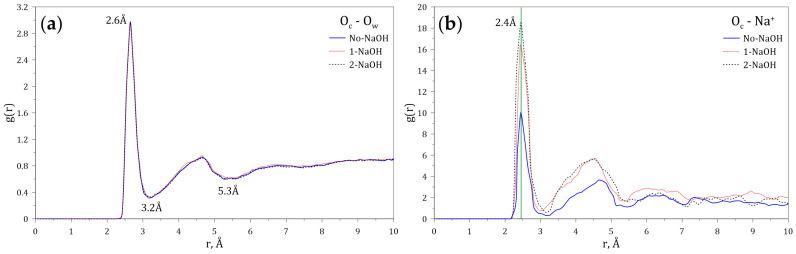
Radial distribution functions (g(r)) of (**a**) O_w_ and (**b**) Na^+^ interacting with the carboxylate group of the HPAM at varying concentrations of NaOH.

Since both the carboxylate and amide groups of the HPAM display polarity, it is expected that hydration shells and electrostatic interactions with ions and molecules may take place. To study these phenomena, the radial distribution functions (RDFs) of both the oxygen (O_w_) atom of the water molecules and the sodium ion (Na^+^) dissolved in water, interacting with the carboxylate group of the HPAM, were calculated at different concentrations of NaOH. Figure 4a shows that the maximum probability of interaction between the O_w_ and the carboxylate group occurs at 2.6 Å, and that the first hydration layer around the carboxylate anion ends at 3.2 Å (3.1 Å [40] and 3.4 Å [41]). A second hydration layer is also observed, which ends at 5.3 Å (5.4 Å [40]). Figure 4b shows that the maximum probability of interaction between Na^+^ and the carboxylate group occurs at 2.4 Å (2.3 Å [40]), indicating that this cation can enter through the first hydration layer located around the carboxylate anion. Furthermore, the variation of the intensities of the peaks located at 2.4 Å indicates that the probability of finding sodium ions around the carboxylate groups increases with the sodium concentration.

**Figure 5:**
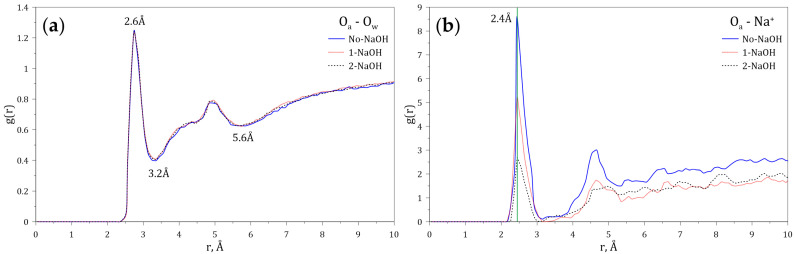
Radial distribution functions (g(r)) of (**a**) O_w_ and (**b**) Na^+^ interacting with the amide group of the HPAM at varying concentrations of NaOH.

Figure 5 shows the RDFs of O_w_ and Na^+^ around the amide group of the HPAM calculated at varying concentrations of NaOH. Figure 5a indicates the presence of a first hydration layer around the amide group ending at 3.2 Å, and of a second hydration layer at 5.6 Å. Figure 5b indicates that the sodium ion (Na^+^) around the amide group is located at a probable distance of 2.4 Å, which also indicates that this cation enters through the first hydration layer. The variation of the intensities of the peaks located at 2.4 Å shows that the presence of sodium around the amide group decreases with sodium concentration.

### 3.3. Interaction of Water with the Face and Edge Surfaces of Molybdenite

**Figure 6:**
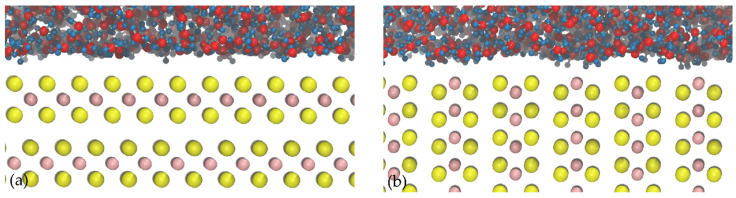
Images of molybdenite/water interfaces: (**a**) molybdenite face; (**b**) molybdenite edge. The atoms’ color codes are as follows: S, yellow; Mo, pink; H, blue; O, red.

Figure 6 shows images obtained from molecular dynamics simulations of water molecules moving near the face and edge surfaces of molybdenite. The space existing between the water molecules and the molybdenite face in Figure 6a suggests the manifestation of the excluded volume effect in this system [42,43]. This phenomenon shows that the interactions between water molecules and the molybdenite basal plane are weak at a molecular level, which explains the hydrophobic character of this surface at a macroscopic level [44,45,46]. On the other hand, Figure 6b shows that water molecules tend to interact with the molybdenite edge through interactions with molybdenum atoms.

**Figure 7:**
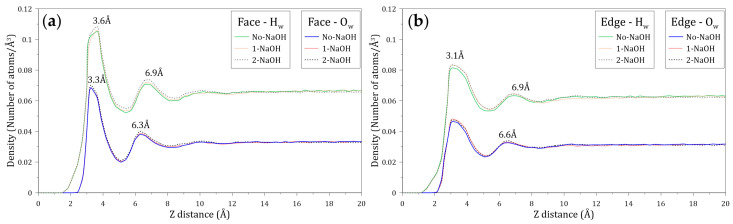
Density profiles of the oxygen (O_w_) and hydrogen (H_w_) atoms of the water molecules located along the Z axis normal to the (**a**) molybdenite face and (**b**) edge surfaces. These calculations were conducted at different concentrations of NaOH.

The distribution of water molecules around the faces and edges of molybdenite was studied by calculating the density profiles of the oxygen (O_w_) and hydrogen (H_w_) atoms of water molecules located along the Z axis normal to these mineral surfaces. Figure 7 shows the atomic density profiles of O_w_ and H_w_ calculated at varying NaOH concentrations. Figure 7a,b indicate that the interactions between the H_w_ and O_w_ atoms and the molybdenite face start around 1.5 and 2.4 Å, respectively, while the interactions of these atoms with the edge arise at 1.2 and 2.0 Å, respectively. As expected, these results indicate that the water molecules tend to be closer to the edge, which agrees with the hydrophilic character of this mineral surface. The presence of the O_w_ and H_w_ atoms is observed at farther distances from the molybdenite faces.

Figure 7a shows that the atomic density profiles of H_w_ and O_w_ around the molybdenite face display initial peaks at 3.6 and 3.3 Å, respectively. The fact that the first peak of O_w_ is located closer to the face surface suggests that the oxygen atoms of the water molecules are preferentially orientated towards the mineral surface in the first hydration layer. The atomic density profiles of H_w_ and O_w_ around the molybdenite face also display secondary peaks at 6.9 and 6.3 Å, respectively. The width of these peaks and their intensity suggest the presence of complex structures of water molecules. The results indicate that the density profiles relax at distances around 10 Å, suggesting a bulk-like behavior.

Figure 7b shows that the atomic density profiles of H_w_ and O_w_ around the molybdenite edge exhibit initial peaks at 3.1 Å, which indicates that there is no preferential orientation of the atoms towards the edge of the molybdenite crystal. The secondary peaks of H_w_ and O_w_ are located at 6.9 and 6.6 Å, respectively. Again, the shape and intensity of these peaks suggest the existence of water structures, and the density profiles also relax at distances around 10 Å. Due to the natural hydrophobicity of the molybdenite faces and the relatively weak hydrophilic character of the mineral edges (in a non-oxidized condition), water molecules tend to be excluded from these surfaces. The primary H_w_ density peaks around the molybdenite face and edge are located at 3.6 and 3.3 Å, distances that are longer than the distance between water molecules bonded by hydrogen bonds, which is about 2.8 Å [47,48,49]. This result demonstrates the weak interaction between the water molecules with the face and edge of molybdenite.

### 3.4. Interactions of the Amide and Carboxylate Groups of the HPAM with the Molybdenite Face

**Figure 8:**
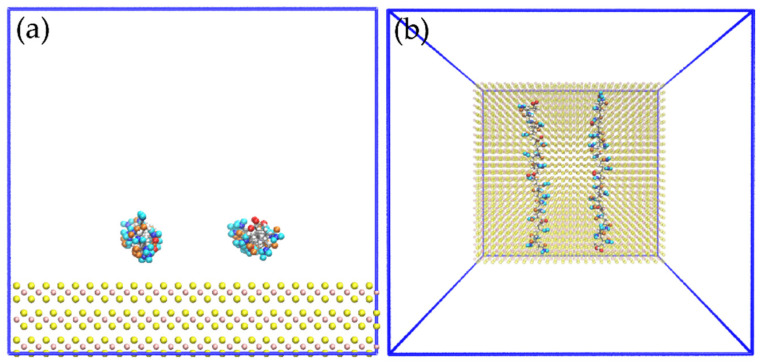
Snapshots of initial configuration for simulation of two molecules of HPAM over the molybdenite face; (**a**) side view and (**b**) top view.

**Figure 9:**
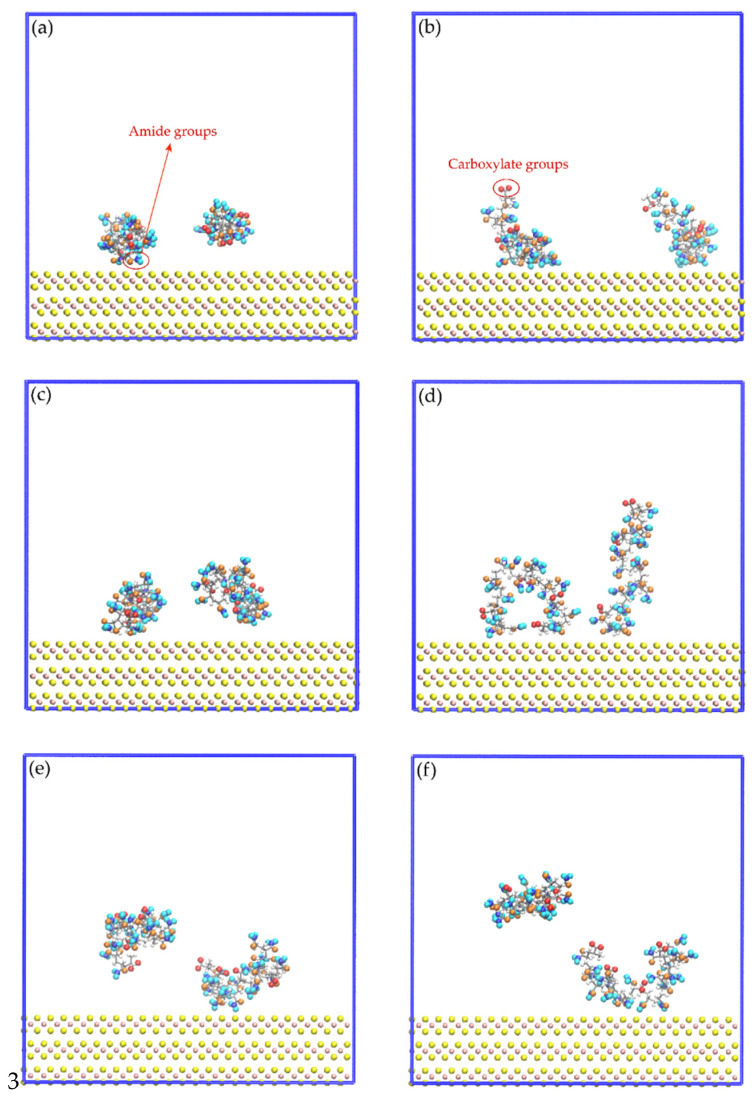
Snapshots of the mineral face/HPAM interfaces: (**a**) No-NaOH, t = 0.1 ns; (**b**) No-NaOH, t = 10.0 ns; (**c**) 1-NaOH, t = 0.1 ns; (**d**) 1-NaOH, t = 10.0 ns; (**e**) 2-NaOH, t = 0.1 ns; (**f**) 2-NaOH, t = 10.0 ns. Na^+^, Cl^−^, and OH^−^ ions are not shown to facilitate the visualization of HPAM molecules.

Figure 8 shows images of the initial configuration of the HPAM molecules in which the carboxylate and amide groups were intended to be directed towards the surface of the molybdenite face. Figure 9 shows snapshots of the HPAM molecules located around the surface of the molybdenite face, at varying simulation times and NaOH concentrations. At short simulation times (0.1 ns), HPAM molecules tend to rearrange themselves in such a way that the amide group moves towards the molybdenite face, and the carboxylate group tends to move away from this hydrophobic surface. As the simulation time elapses, the hydrophobic interactions of the face surface with the amide group of the HPAM molecule become more relevant.

**Figure 10:**
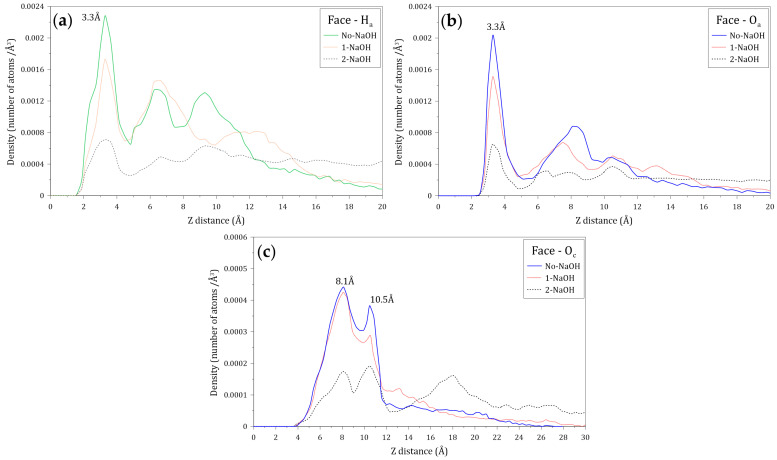
Density profiles of the (**a**) hydrogen atoms of amide groups (H_a_), (**b**) oxygen atoms of amide groups (O_a_), and (**c**) oxygen atoms of carboxylate groups (O_c_) located along the Z axis normal to the molybdenite face. These calculations were conducted at different concentrations of NaOH.

Figure 10 shows the atomic density profiles of the hydrogen (H_a_) and oxygen (O_a_) atoms of the amide group, and the oxygen (O_c_) atom of the carboxylate group in the vicinity of the molybdenite face, calculated at different NaOH concentrations. Figure 10a,b indicate that the interactions between the H_a_ and O_a_ atoms and the molybdenite face start at around 1.5 and 2.7 Å, respectively. Figure 10a shows that the first peak of H_a_ is displayed at 3.3 Å from the surface, which indicates that most of these atoms interact at this distance with the molybdenite face. There are several H_a_ peaks beyond 3.3 Å, which reveals the presence of other layers of HPAM molecules. It is worth noting that the intensity of the different peaks decreases in the presence of NaOH molecules, indicating some effect of the ions on the HPAM adsorption on the face. Figure 10b shows the atomic density profiles of the O_a_ atoms, which display a sharp peak around 3.3 Å, and that the atomic density decreases with the increase of the NaOH concentrations. Figure 10c shows the atomic densities of the O_c_ atoms that show two major peaks at 8.1 and 10.5 Å, which indicates that the interactions between the molybdenite face and the carboxylate group are weaker than those with the amide group. It can also be seen that the interactions of the carboxylate group with the mineral face start at 3.9 Å. A substantial reduction of the peak intensities can be seen when 2 NaOH molecules are added, causing the highest number of O_c_ atoms to be located 10.5 Å from the molybdenite face, which indicates that the increase in NaOH causes the carboxylate groups of the flocculant to move away from the surface of the mineral face. All these results indicate that the interaction between the HPAM and the molybdenite face occurs mainly through the interaction of the amide group with the basal planes of the mineral.

### 3.5. Interactions of the Amide and Carboxylate Groups of the HPAM with the Molybdenite Edge

**Figure 11:**
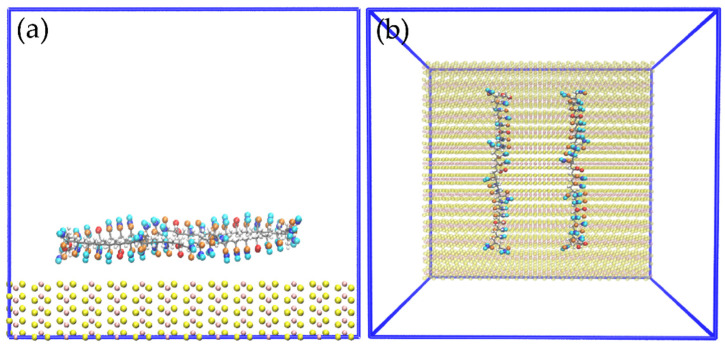
Snapshots of the initial configuration for simulation of two molecules of HPAM over the edge of MoS_2_: (**a**) side view, (**b**) top view.

**Figure 12:**
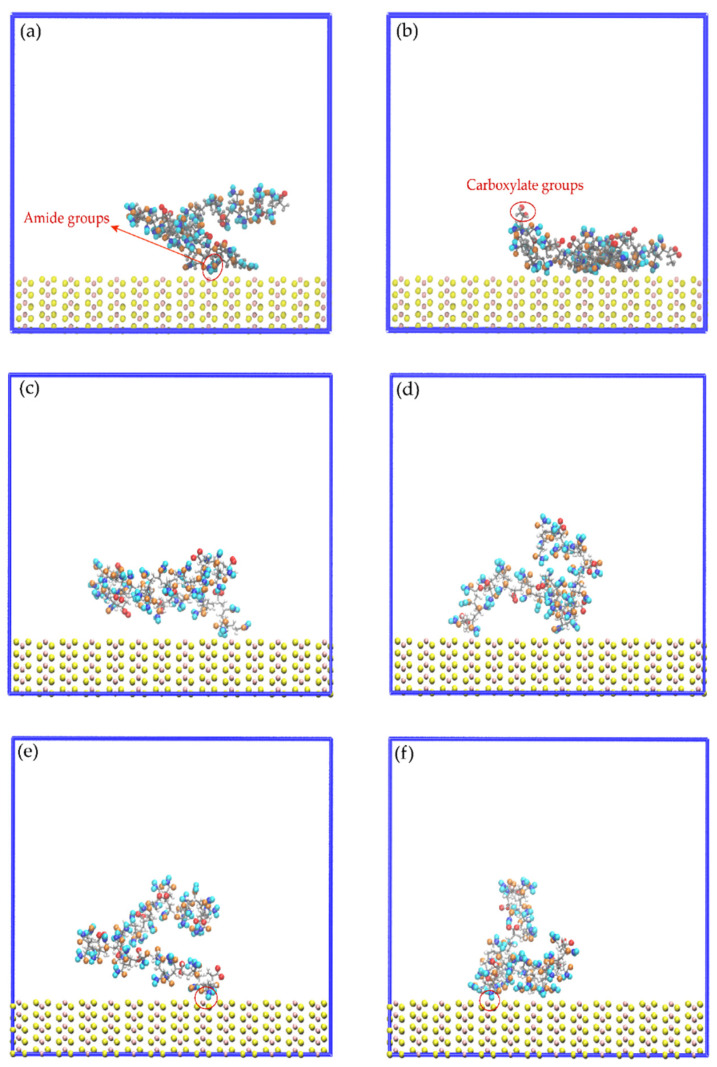
Snapshots of the mineral edge/HPAM interfaces: (**a**) No-NaOH, t = 0.1 ns; (**b**) No-NaOH, t = 10.0 ns; (**c**) 1-NaOH, t = 0.1 ns; (**d**) 1-NaOH, t = 10.0 ns; (**e**) 2-NaOH, t = 0.1 ns; (**f**) 2-NaOH, t = 10.0 ns. Na^+^, Cl^−^, and OH^−^ ions are not shown to facilitate the visualization of HPAM molecules.

Figure 11 displays images of the initial configuration of the HPAM molecules with the carboxylate and amide groups directed towards the surface of the molybdenite edge, while Figure 12 shows snapshots of the HPAM obtained at varying simulation times and NaOH concentrations. Again, at longer simulation times, HPAM molecules reorient in such a way that the amide group move towards the molybdenum atoms exposed on the edge surface, and the carboxylate groups tend to organize away from the edge. The interaction of the amide group with the molybdenite edge becomes stronger with time.

**Figure 13:**
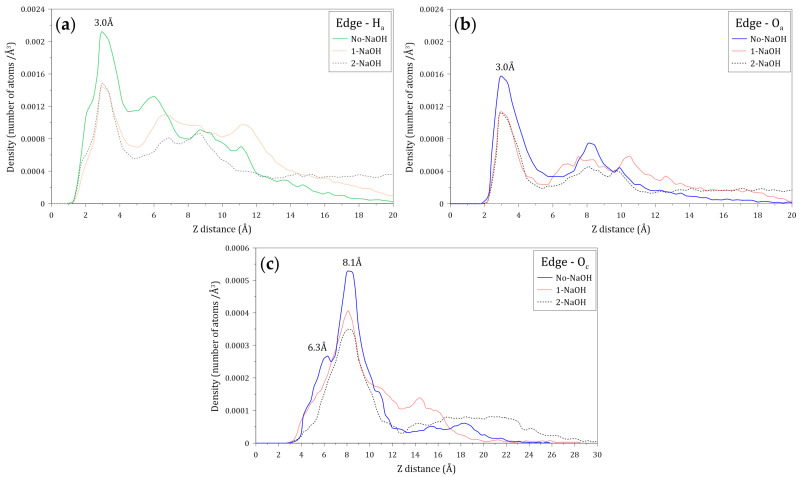
Atomic density profile of (**a**) hydrogen atoms of amide groups, (**b**) oxygen atoms of amide groups, and (**c**) oxygen atoms of carboxylate groups of the flocculant on the molybdenite edge surface.

Figure 13 shows the atomic density profiles of the H_a_, O_a_, and O_c_ atoms around the molybdenite edge, calculated at varying NaOH concentrations. Figure 13a,b indicate that the interactions between the H_a_ and O_a_ atoms and the molybdenite edge start around 1.2 and 1.8 Å, respectively, which are shorter distances than those observed with the molybdenite face. As seen in Figure 13a, the first H_a_ density peak is displayed at 3.0 Å, with the intensity decreasing in the presence of the NaOH molecules which indicates that HPAM adsorption on the edges is negatively affected by the presence of sodium and hydroxide ions. Figure 13b shows the atomic density profiles of the O_a_ atom, which display a sharp peak at around 3.0 Å; the atomic density of this peak decreases with the increase of NaOH. This result indicates that the number of oxygen atoms of the amide group decreases in the vicinity of the molybdenite edge because of the increase in NaOH. The atomic densities of the O_c_ atoms presented in Figure 13c show two major peaks at 6.3 and 8.1 Å, which indicates that the interactions between the molybdenite edge and the carboxylate group are weaker than those with the amide group. It can also be seen that the interactions of the carboxylate group with the mineral edge start at 3.0 Å. The peak intensities decrease in the presence of NaOH molecules, which indicates that the increase in NaOH causes the carboxylate groups of the flocculant to move away from the surface of the mineral face.

## 4. Conclusions

The main conclusions of the study are summarized as follows:

- The results obtained from molecular dynamics simulations show that the interactions between the HPAM and molybdenite are mainly explained by the interactions of the amide group with the faces and edges of molybdenite.
- Molecular dynamics simulations show that the HPAM molecule rearranges, the amide group moves towards the molybdenite face or edge, and the carboxylate group moves away from the mineral.
- The results obtained in the simulations show that the interactions of the HPAM with the molybdenite edge are slightly stronger than the interaction of this molecule with the mineral face.
- Simulations indicate that the presence of the sodium and hydroxide ions reduces the concentration of HPAM around the face and edge surfaces. HPAM adsorption on molybdenite is expected to be affected at high pH and in salty water.
- The density profiles of the oxygen (Ow) and hydrogen (Hw) atoms of the water molecules located along the Z axis normal to the face and edge surfaces of molybdenite indicate that the water molecules tend to be closer to the edge, which confirms the hydrophilicity character of this mineral surface observed at a macroscopic level.
- The conclusions obtained through molecular dynamic simulations are in line with the results obtained in previous studies completed at a macroscopic level, which indicated that HPAMs adsorb on molybdenite particles and reduce their hydrophobicity.

## Figures and Tables

**Table 1:** Force field parameters for molybdenite (MoS_2_).

Mineral	Atom	ɛ (Kcal/mol)	rm (Å)	Mulliken Charge (q)
Molybdenite (MoS_2_)	Mo	0.056	3.056	0.458
	S	0.274	4.035	−0.229

## Data Availability

The data presented in this study are available on request from the corresponding author.
